# Growth and toxicity of *Halomicronema metazoicum* (Cyanoprokaryota, Cyanophyta) at different conditions of light, salinity and temperature

**DOI:** 10.1242/bio.043604

**Published:** 2019-10-15

**Authors:** Mirko Mutalipassi, Valerio Mazzella, Giovanna Romano, Nadia Ruocco, Maria Costantini, Francesca Glaviano, Valerio Zupo

**Affiliations:** 1 Marine Biotechnology Department, Stazione Zoologica Anton Dohrn, Villa Comunale, 80121, Napoli, Italy; 2 Integrative Marine Ecology Department, Benthic Ecology Centre, Stazione Zoologica Anton Dohrn, Punta San Pietro, 80077 Ischia, Italy

**Keywords:** Cyanobacterium, Toxins, Environment, Sea urchin, Rotifers

## Abstract

Cyanobacteria may live in the water column and in the benthos of aquatic environments, or be symbionts of other organisms, as in the case of *Phormidium*-like cyanobacteria, known to influence the ecology of freshwater and marine ecosystems. A strain of *Phormidium*-like cyanobacteria has been recently isolated as a free-living epiphyte of leaves of *Posidonia oceanica* (L.) Delile in the Mediterranean sea and its biology and ecology are herein investigated. It was identified as *Halomicronema metazoicum*, previously known uniquely as a symbiont of marine sponges. We cultivated it in a range of light irradiances, temperatures and salinities, to establish the most suitable conditions for the production of allelopathic and toxic compounds. The bioactivity of its spent culture medium was measured by means of standard toxicity tests performed on two model organisms. Our results indicate that at least two bioactive compounds are produced, at low and high irradiance levels and at two temperatures. The main compounds influencing the survival of model organisms are produced at the highest temperature and high or intermediate irradiance levels. The present research contributes to the understanding of critical toxigenic relationships among cyanobacteria and invertebrates, possibly influencing the ecology of such a complex environment as *P. oceanica*. Future isolation, identification and production of bioactive compounds will permit their exploitation for biotechnologies in the field of ecological conservation and medical applications.

## Abstract (precis)

**Summary:** This is a contribution to understand toxigenic relationships between cyanobacteria and invertebrates because such interactions, still largely unexplored, may dramatically influence the ecology of several species associated with seagrass ecosystems.

## INTRODUCTION

Photosynthetic cyanobacteria, also known as ‘blue-green algae’, are distributed worldwide in any photic and moist environment (Gaylarde et al., 2004), including marine waters, freshwaters, natural grounds and extreme environments (Dahms et al., 2006). These prokaryotes, living either as unicellular or colonial forms, exhibit a remarkable taxonomic diversity (Whitton, 1992; Usher et al., 2004) and an even more notable functional diversity (Barberousse et al., 2006). As a matter of fact, physiologic differences among strains may largely encompass the dissimilarities among species and genera (Pfeiffer and Palińska, 2002), and their noteworthy diversity also explains the importance as potential producers of novel bioactive substances with economic potential (Shimizu, 2003; Blunt et al., 2006). A long tradition of screening and separation of active biomolecules took advantage of the secondary metabolites produced by cyanobacteria to develop antifouling agents (Abarzua et al., 1999), antibiotics (Bloor and England, 1989), sunscreens (Böhm et al., 1995), antimycotics (Bonjouklian et al., 1991) and a plethora of useful applications (Burja et al., 2001). Such an abundance of uses is due to the variety of secondary metabolites synthesized, ranging from carotenoids and phycobiliprotein pigments – having commercial value as feed additives and colour enhancers for foods (Shimizu, 2003) – to toxic polysaccharides used as pesticides or in clinical applications (Kulik, 1995). Various cyanobacteria also produce vitamins of the B and E complexes (Plavšić et al., 2004) and they are exploited for large-scale productions in special photobioreactors.

Cyanobacteria also play key ecological roles (Pietsch et al., 2001) and exhibit a wide diffusion thanks to their aptitude to colonize any habitat, and produce active biomolecules reaching the environment by simple leaching from their mattes (Jüttner et al., 2001). Other secondary metabolites are wound-activated by grazers (Manivasagan et al., 2017). They were demonstrated to control the presence of other organisms in benthic environments (Borges et al., 2015), as well as in planktonic environments (Dias et al., 2017) and influence the quality of waters destined for human consumption (Jüttner et al., 2001), when diffused in freshwater basins.

However, cyanobacteria may be also symbiotically associated with animals and algae. For example, their association with demospongiae is quite frequent in the marine environment (Caroppo et al., 2012), as well as with corals and other invertebrates (Taylor et al., 2007). Recent findings indicate that a species of cyanobacteria, *Halomicronema metazoicum*, may be both a symbiont of marine sponge and a free-living organism associated to leaves of the seagrass *Posidonia oceanica* (Ruocco et al., 2019). The host sponge *Petrosia ficiformis* exhibited haemolytic activity and influenced brine shrimp vitality and sea urchin development (Pagliara and Caroppo, 2010, 2011) and these activities were supposed to be mediated by symbiotic cyanobacteria strains (Caroppo et al., 2012). These cyanobacteria were previously assigned to the genus *Phormidium* (Geitler 1932) according to the botanical code, and to the *Lyngbya/ Plectonema/Phormidium*-group B (Rippka et al., 1979) according to the bacteriological system.

Cyanobacteria with *Phormidium*-like morphologies do not form a monophyletic group (Giovannoni et al., 1988), but their collective ability to produce toxic exudates is well known (Dias et al., 2017; Wood et al., 2017). The new species *H. metazoicum* was established to classify non-heterocystous, thin filamentous symbiotic cyanobacteria (Caroppo et al., 2012) and further studies (Ruocco et al., 2019) indicated that this species may also live as an epiphyte of *P. oceanica* leaves. Given the known complexity of interactions among plant and animal communities associated to seagrass leaves (Mazzella et al., 1991), and the ability of cyanobacteria to produce allopathic compounds (Dias et al., 2017; Devlin et al., 1977), their presence may likely influence life and evolution of various organisms.

Proliferations of benthic mat-forming *Phormidium* have been reported in various sites and they commonly produce a range of neurotoxins, collectively known as anatoxins, prompting risks to human and animal health (Dias et al., 2017). In addition, *Phormidium*-like cyanobacteria are known to produce a range of natural toxins including anatoxin-a, homoanatoxin-a, microcystins, portoamides and saxitoxins (Borges et al., 2015; Gugger et al., 2005; Teneva et al., 2005; Kouzminov et al., 2007). Since their presence may produce acute toxicity for animals and humans (Wood et al., 2017) and their natural blooms correspond to deadly conditions for various organisms in the same communities (Shurin and Dodson, 1997), there is rising awareness of the risks driven by *Phormidium* proliferations (Catherine et al., 2013; Echenique-Subiabre et al., 2016).

The production of toxic compounds is often modulated by salinity, light irradiance and temperature (Caroppo et al., 2012) and the aim of this study was to characterize the environmental conditions maximizing the production of allochemicals and toxins produced by *Phormidium*-like cyanobacteria (Dias et al., 2017). We also aim at defining sensible experimental tools to detect and measure their toxicity, to identify possible influences on organisms in the leaf stratum of Mediterranean seagrasses. Since previous tests revealed toxic compounds naturally released in the water by similar cyanobacteria (Dias et al., 2017), and preliminary research confirmed the toxicity of *H. metazoicum* on *Artemia salina* nauplii (Zupo et al., 2019), we investigated the toxicity of natural exudates in a range of environmental conditions. A strain of *H. metazoicum* isolated from *P. oceanica* leaves (Ruocco et al., 2019) has been cultivated in the laboratory in three conditions of light irradiance, temperature and salinity, and its natural harmfulness has been assayed on model organisms using standard toxicity tests (Dahms et al., 2011). Since the relevance of culturing cyanobacteria also derives from their ability to produce compounds for biotechnological applications (Moore et al., 1988; Gerçe et al., 2009), this investigation will improve our ability to maximise the production of bioactive metabolites (Sivonen and Börner, 2008; Raniello et al., 2007).

## RESULTS

**Fig. 1.**
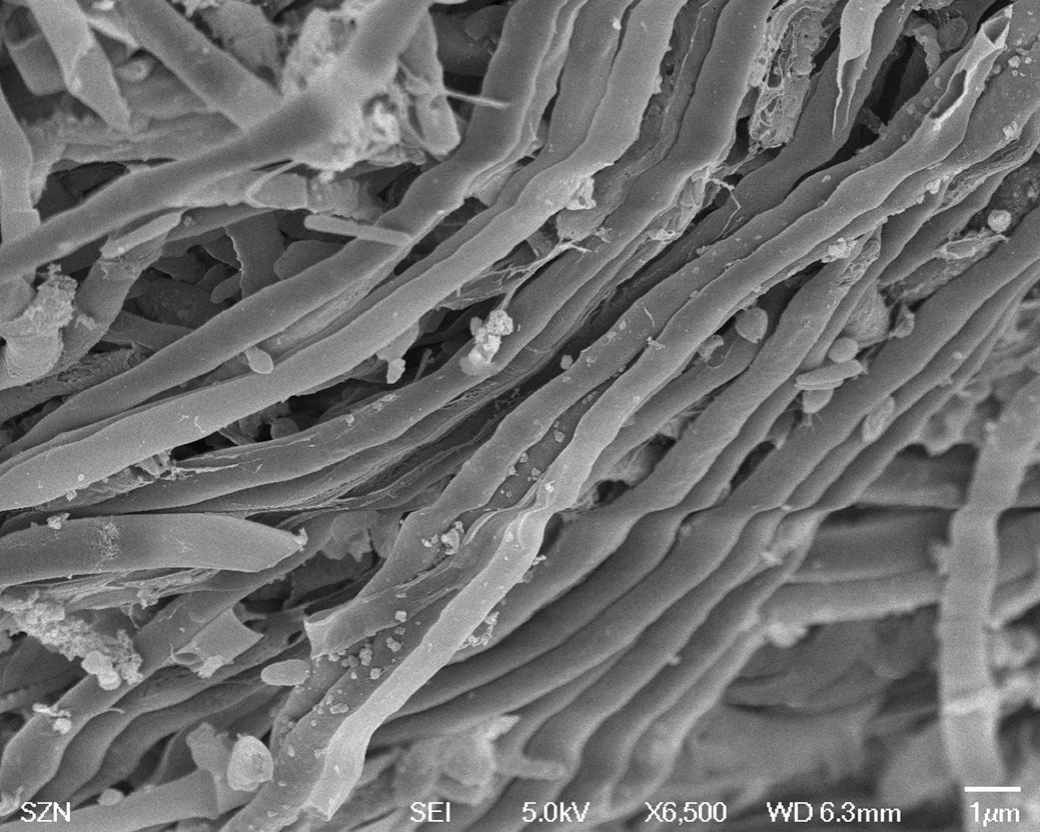
**Scanning Electron Microphoto of a sample of cyanobacteria showing a dense matte of non-heterocystous thin filaments.** Some small vesicles of amorphous exudates are present on their surface.

**Fig. 2.**
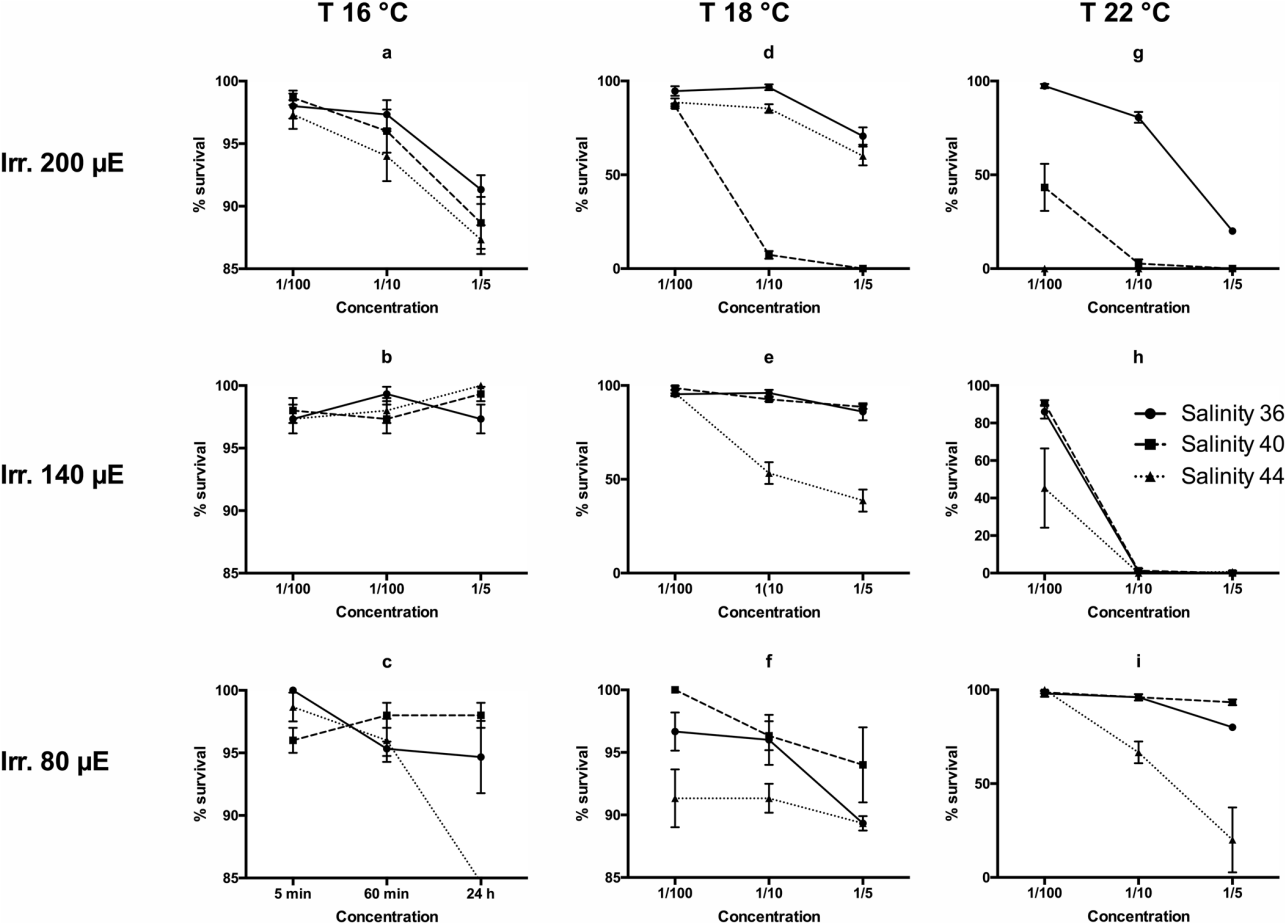
**Survival rates of *B.plicatilis* recorded after 24 h of exposure to three concentrations of the spent culture medium of *H.metazoicum*, cultivated at three temperatures, three irradiance levels and three salinities.**

The strains of *H.* *metazoicum* isolated and cultured for the purposes of this study appeared clean of contaminants and shaped as dense mattes of non-heterocystous, thin filaments (Fig. 1), containing small aggregates of mucous exudates. The spent culture medium, after 40 d of growth, appeared brownish but transparent. Various culture conditions produced complex patterns of responses in *Brachionus plicatilis*, according to the time of exposure, temperature, irradiance and salinity. Negative controls (containing fresh *f/2* medium at the corresponding concentrations, as above specified) exhibited an almost constant number of individuals during the experiment and, after 24 h, the survivorships were still 94% (±7.21), while the survival rates accounted for 100% both 5 and 60 min after the start of the experiment. Overall, the time of exposure did not produce an evident effect among treatments (ANOVA, *P*>0.05). Only, in a few conditions (e.g. at salinity 40, irradiance 80 µE and temperature 22°C) there was a significant difference between the records obtained at 24 h and those obtained at 5 and 60 min (*P*<0.01). In addition, the records obtained at 5 and 60 min exhibited no significant differences between them, when analysed by Wilcoxon test and in most cases there was no effect on the survival rates compared with those of negative controls (survival close to 100%). For this reason it is useful to analyse the results obtained at 24 h, exhibiting the largest differences (Fig. 2). Survival rates at the highest irradiances decreased in most treatments in a dose-dependent manner, with higher slopes between the concentrations 1:100–1:10 and the highest mortalities recorded between 1:10−1:5 (Fig. 2D,G,H). The factors mainly influencing the differences in mortality rates were temperature and irradiance. Salinity produced significant differences (ANOVA, *P*<0.01) at 18°C and 22°C, especially at the lowest (Fig. 2F,I) and the highest (Fig. 2D,G) irradiance levels. The highest temperatures (Fig. 2G–I), salinities and irradiances (Fig. 2D,G) represent the conditions maximizing the production of toxic compounds. Low temperatures (Fig. 2A–C) and low irradiances (Fig. 2C,F,I) produced media having scarce or null toxigenic effects on *B. plicatilis*.

**Fig. 3.**
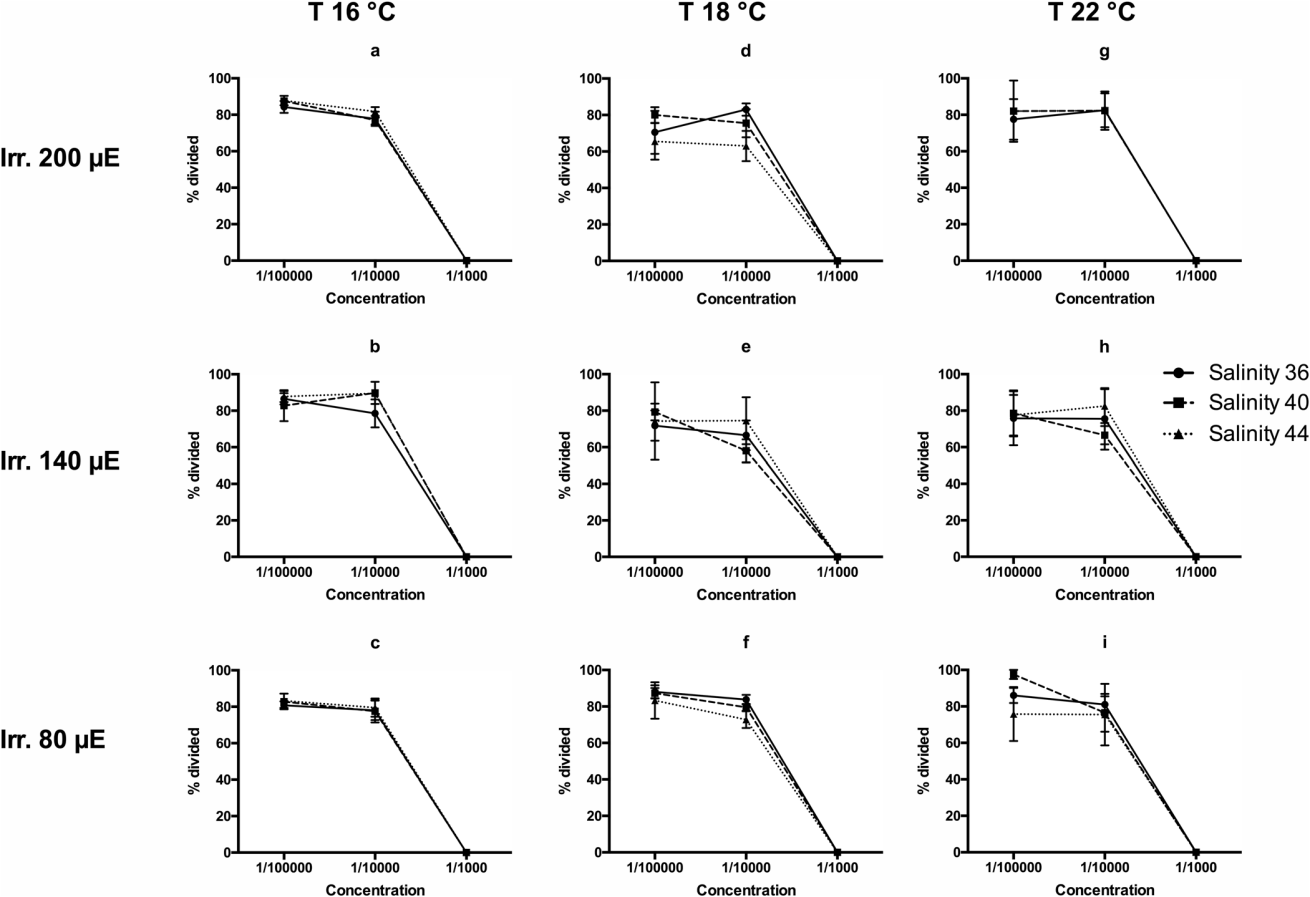
**Rates of first divisions of embryos of *P.lividus* recorded after 1 h of exposure to three concentrations of the spent culture medium of *H.metazoicum*, cultivated at three temperatures, three irradiance levels and three salinities.**

In the case of sea urchin embryos, various development phases offered different results. Negative controls produced 99.0% (±0.4) of divided embryos, recorded 1 h after the *in vitro* fertilization of eggs. In contrast, in all treatments, regardless of salinity, temperature and irradiance (ANOVA, *P*>0.05) the concentration 1:1000 blocked the development at the first division (Fig. 3), while the effect of lower concentration was low or null.

**Fig. 4.**
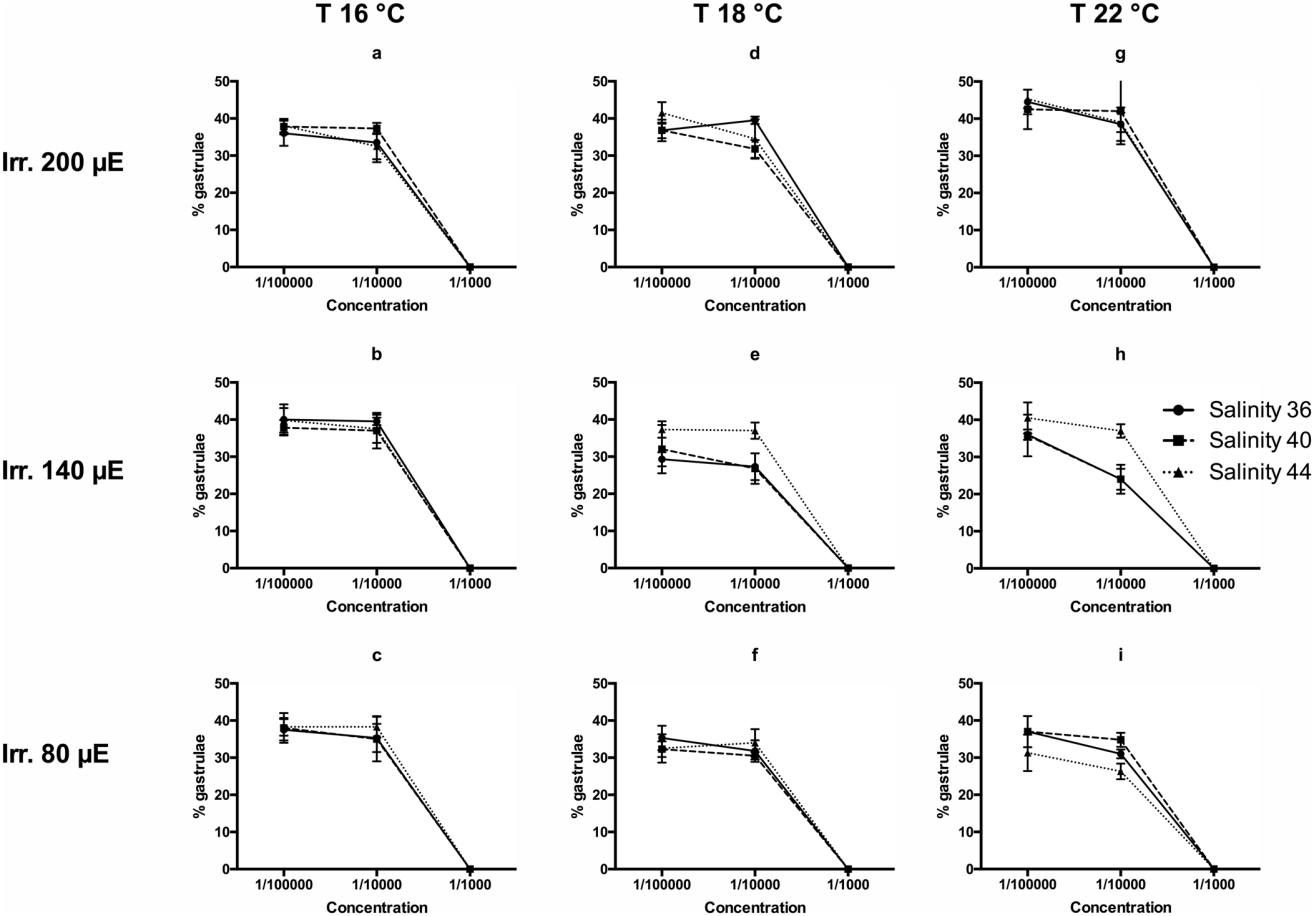
**Rates of gastrulation of embryos of *P.lividus* recorded after 8 h of exposure to three concentrations of the spent culture medium of *H.metazoicum*, cultivated at three temperatures, three irradiance levels and three salinities.**

**Fig. 5.**
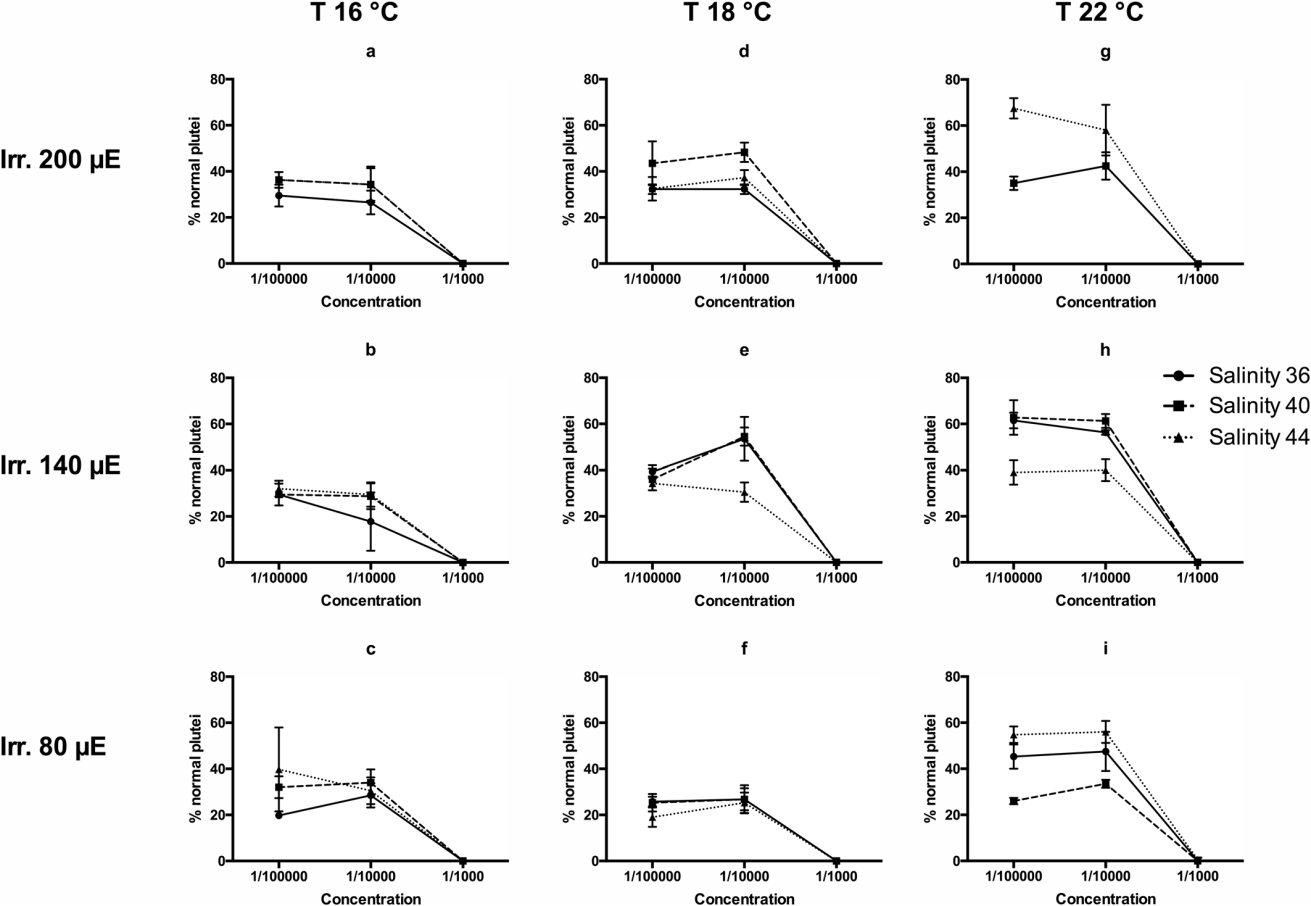
**Rates of production of normal plutei of *P.lividus* recorded after 48 h of exposure to three concentrations of the spent culture medium of *H.metazoicum*, cultivated at three temperatures, three irradiance levels and three salinities.**

The development of sea urchin embryos to gastrulae, passing through the stage of blastulae, offered a complex array of results (Fig. 4). Also in this case, a threshold was represented by the concentration 1:1000, blocking or retarding the development, while the concentrations 1:10,000 and 1:100,000 produced results not significantly different from those exhibited by negative controls. The effect of salinity on gastrulation was generally not significant, with a few differences in various treatments (e.g. Fig. 4E,H,I). As well, the patterns of development to normal plutei were complex, but confirmed the efficacy of the highest concentration (Fig. 5). In this case, the maximum efficacy was recorded at the lowest temperature (16°C) and the lowest irradiance, generating the lowest percentages of normal plutei (Fig. 5A–C,F). Salinity showed contrasting results at the highest temperature. The effective dose was consistently between 1:10,000 and 1:1000.

## DISCUSSION

The grow-out of cyanobacteria was continuous in our experimental conditions (Ruocco et al., 2019) and the cultures were free of contaminants. The absence of other organisms was likely due to toxic compounds naturally produced by cyanobacteria (Dias et al., 2017), having an allopathic effect on bacteria, protozoans and algae (Snell and Carmona, 1995; Hughes et al., 1958). The same compounds were active on the rotifer (Preston and Snell, 2001) at a concentration comprised between 1:10 and 1:5. The effects were slightly increasing upon time but in most treatments they were evident after 24 h, indicating an acute toxicity clearly affecting the vitality and the survival of tested organisms. Trends of toxicity were consistent among treatments at various times and we chose to take into account the final readings at 24 h, to simplify the evaluation of the median lethal concentration (Dahms and Hellio, 2009).

The results of toxicology tests performed on rotifers were quite reproducible and the differences among replicates were generally low (Suga et al., 2007). However, the patterns of responses according to salinity were puzzling, if various irradiances and temperatures were compared. In general, the maximum efficacy was reached at intermediate irradiance (140 µE) and higher temperatures (18–22°C) and salinities (40–44). However, the highest salinity was consistently effective at lower (80 µE) irradiance while intermediate salinity (40) was effective mainly at the highest irradiance (200 µE). The production of toxic exudates was maximum at 22°C, 140–200 µE and salinity 44, producing total mortality at a concentration of 1:10. The temperature is a critical factor because the toxicity was lowest in cultures cultivated at 16°C. Interestingly, at the salinity characterizing most oceans (36 psu) the highest irradiance (200 µE) induced a decrease in the toxicity of cyanobacteria. However, the result of these acute toxicology tests measure the effects of given compounds in specific experimental conditions (Preston et al., 1999) and we cannot exclude that various families of bioactive compounds (Raniello et al., 2007), having different effects over longer times of exposure, are produced by the same strain.

For this reason it is useful to compare the results with those obtained in standard toxicology tests performed on sea urchin embryos (Romano et al., 2003). In this case, toxic compounds appear to affect selected phases of embryo development. The first division is strongly affected by the presence of cyanobacteria toxins at a concentration comprised between 1:100,000 and 1:10,000 with no reference to salinity, irradiance or temperature. In fact, all experimental conditions produced high percentages of divided embryos at 1:100,000 and total block of embryo development at 1:10,000. A slight increase of toxicity was observed at the highest temperature (22°C) and the highest salinity (44), coherently to what observed in *B. plicatilis* tests, but the differences in this case are scarcely significant. Similar trends were observed in the influences on the gastrulation process, since the strongest effects were triggered by the media obtained at the highest temperature (22°C), especially when coupled with intermediate and highest salinities (40–44) and low or medium irradiances (80–140 µE). Thus, a different class of compounds could be responsible for this activity, or the changed metabolism of embryos in this phase could be influenced by different compounds, in the range of metabolites produced by cyanobacteria. The patterns were totally inverted when the development of plutei was considered. In this case, in fact, the lowest percentages of normal plutei were triggered by spent medium collected at the lowest temperature (16°C) regardless of irradiance and salinity. In contrast, the highest temperature (22°C) triggered similar effects only at the highest (44) or intermediate (40) salinity, in accordance with *B. plicatilis* bioassays. We conclude that the compounds influencing the development of larvae, mainly produced at lower temperatures, are different from those influencing the mortality of *B. plicatilis* and the development of the sea urchin until the gastrula stage, mainly produced at the highest temperatures and intermediate or low irradiances. In contrast, the first division of sea urchin embryos appears to be a delicate process, blocked at the same rate by compounds produced in any condition of light, temperature and salinity.

Interestingly, the median lethal concentration (Boudou and Ribeyre, 1989) of allopathic compounds produced at higher temperature (22°C), medium irradiance (140 µE) and medium-high salinities, was different in *B. plicatilis* and sea urchin embryos. The latter reacted at concentrations of the spent medium at least 2 orders of magnitude lower than those active on rotifers. A second class of compounds could be produced at low temperature (16°C) without distinction of irradiance and salinity, and it influenced the process of development of sea urchin plutei at very low concentrations, comprised between 1:100,000 and 1:10,000. These compounds produced at low-temperature influenced *B. plicatilis* at concentrations as high as 1:5, but we still ignore both their concentration in the spent medium and their chemical nature.

We must consider, however, that proteins and polypeptides (Zhang et al., 2011) are among the most important toxic compounds produced by cyanobacteria and it is known that changes in environmental conditions (e.g. salinity, pH and temperature), modify their structure and may cause the formation of cytotoxic protein aggregates, with the synthesis of ‘stress’ proteins (Gross, 2004). Portoamides, for example, are cyclic peptides having a clear allopathic activity and an antiproliferative effect on human lung-carcinoma cells (Ribeiro et al., 2017). They are among the most interesting compounds produced and released by *Phormidium*-like cyanobacteria. The differences in toxicity detected at different salinities and temperatures are in line with previous findings (Chorus et al., 2000) and indicate that cyclic polypeptides (e.g. microcystins and portoamides), typically produced by these organisms, could be the main products influencing the survival of different model organisms, at corresponding median lethal concentrations.

In addition, some families of microcystins are known to induce apoptosis in various organisms and these compounds, produced at low temperatures, could be responsible for the irregular development of plutei. Microcystins can also trigger cytoskeleton disruption in human hepatocytes (Falconer and Yeung, 1992), after disorganization of cytoplasmic microtubules, cytokeratin intermediate filaments and actin microfilaments (Ding et al., 2000). They were demonstrated to produce oxidative stress, exposing cells to the activity of reactive oxygen species (ROS). For example, the exposure to microcystins causes oxidative stress in Sertoli cells of rats, through decreased antioxidative enzyme activity and increased ROS activity (Yi et al., 2011). Oxidative stress and apoptosis are related processes and the production of ROS has been suggested to be involved in programmed cell death under various conditions, including chemical injury (Buttke and Sandstrom, 1994; Tan et al., 1998; Kannan and Jain, 2000). Thus these compounds, produced at higher temperatures and irradiances, could be responsible for the block of cell divisions in sea urchin embryos.

These compounds might reveal interesting biotechnological applications even in the field of human medicine (Thompson, 1995) since they might have antitumor activity against some cell lines. Indeed, sea urchin embryos have been successfully used to test the antimitotic activity of candidate compounds for chemotherapy, in oncological research (Leite et al., 2012), and the induction of blockage at the first mitotic division might suggest a similar antimitotic effect on fast dividing tumour cells (Gutierrez, 2016). Different tissues demonstrated variable responses to microcystins (Yi et al., 2011) and a recent study (Zhang et al., 2011) demonstrated that the expression level of p53 increased when human Sertoli cells were exposed to microcystins, suggesting that they induce apoptosis by modulating the expression of p53, as well as modulating the expression of Bcl-2 proteins. Hence, some of the putative compounds responsible for the observed toxic effects play key roles in various mechanisms involved in the apoptotic pathways. In particular, p53, bcl-2, bax and caspase-3 are probably involved in cyanobacteria-induced cell damage and toxicity (Dias et al., 2017), and further investigations on the toxicological role of cyanobacterial products in apoptosis-related signalling pathways (Ribeiro et al., 2017) will clarify the nature, the specificity and the mechanism of action of the compounds produced at low and high temperatures by *H.* *metazoicum*.

## MATERIAL AND METHODS

### Collection of cyanobacteria samples

Cyanobacteria mattes were collected in spring from leaves of *P.* *oceanica*, in a meadow off Lacco Ameno d'Ischia (Bay of Naples, Italy, 40°44′56″ N, 13°53′13″ E), using sterilized forceps. They were transferred to multi-well dishes filled with 4 ml of *f/2* medium. Cultures were renovated various times up to a complete purification. Morphological analyses and molecular tools (Ruocco et al., 2019) were applied and a free-living strain of *H.* *metazoicum* was identified. No contamination was detectable under both light microscopy and SEM in exponentially growing cultures of this uni-cyanobacterial culture, even under high magnification, probably due to the antibiotic properties attributed to their exudates (Dias et al., 2017). Pure strains were cultured in *f/2* medium in 400 ml glass dishes kept in a thermostatic chamber at a temperature of 18°C, with light irradiance of about 200 µE and 12/12 h dark/light photoperiod. The medium was renovated every 15 days to avoid changes of culture conditions due to evaporation.

### Production of spent medium

Small portions of cyanobacteria mattes were collected from mother cultures, cut in pieces of about 5 g (fresh weight) and individually cultured in 2 l Erlenmeyer flasks containing 1.5 l of *f/2* medium. Two replicate flasks were cultivated under each condition of light, temperature and salinity, according to a factorial experimental plane (Table 1) containing 27 combinations. In particular, we tested conditions of salinity, temperature and irradiance that could be found in various habitats of the Mediterranean sea and combined them to test the production of bioactive compounds. Cyanobacteria were cultivated 40 days in these conditions, to facilitate an accumulation of secondary metabolites in the medium. Spent culture media were collected at the end of the production period, filtered over a 0.22 µm Millipore filter, stored in glass vessels and kept at −20°C up to the start of bioassays.

**Table 1.**
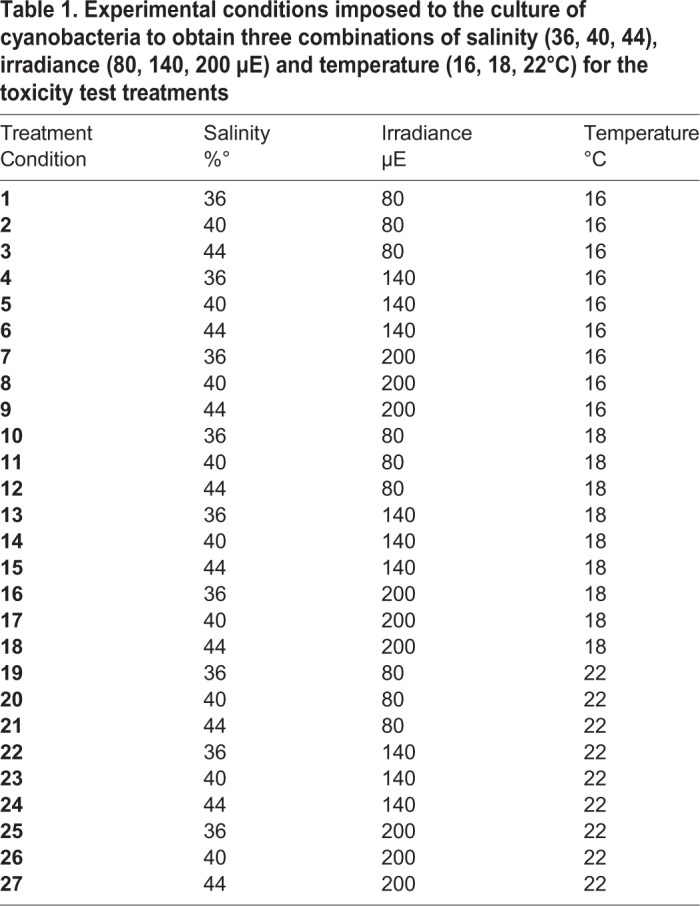
**Experimental conditions imposed to the culture of cyanobacteria to obtain three combinations of salinity (36, 40, 44), irradiance (80, 140, 200 µE) and temperature (16, 18, 22°C) for the toxicity test treatments**

### Preparation of toxicity tests

Toxicity tests were performed on two model organisms, to compare the results on taxa exhibiting a different resistance to toxic pollutants and, in particular, on adults of the rotifer *B.* *plicatilis* (Snell and Carmona, 1995) and embryos of the sea urchin *Paracentrotus lividus* (Romano et al., 2003)*.* The contents of the above described two replicate flasks produced for each culture condition, were pooled prior to the tests. The spent medium was sampled and diluted at various concentrations in filtered seawater, according to the sensitivity of target models. Basically, we took into account the following six dilutions of the culture medium in filtered seawater: 1:5, 1:10, 1:100, 1:1000, 1:10,000, 1:100,000 in volume. However, in the case of *P. lividus*, the highest concentrations (1:5, 1:10, 1:100) were not considered in further analyses, because they produced immediate mortality of sensitive embryos (Romano et al., 2003). In the case of *B. plicatilis* the lowest concentrations (1:1000, 1:10,000, 1:100,000) were not considered in further analyses, because they did not produce any effect on this stronger organism, able to partially detoxify poisons (Dahms et al., 2011).

### Toxicity tests on *B. plicatilis*

A single clone of *B. plicatilis*, maintained in continuous culture at the Stazione Zoologica Anton Dohrn, has been used to perform tests on rotifers. This clone continuously produces offspring when cultivated at 20°C and it is fed on cultures of *Dunaliella* sp. replaced every 5 days. As referred to above, three concentrations of spent medium were employed for bioassays on *B. plicatilis*, i.e. 1:5, 1:10 and 1:100 in volume, after preliminary tests indicating concentrations lower than 1:100 did not produce any effect in respect to controls (Dahms et al., 2011). Fifty adult individuals of *B. plicatilis* were transferred into three replicates of 5 ml multi-well plates filled with 4 ml of solution (spent medium diluted in seawater) for each of the above-mentioned concentrations, and their motility and survival rates were checked at 5 min, 60 min and 24 h, for each of the experimental conditions.

### Toxicity tests on *P. lividus* embryos

Three dilutions of spent medium were assayed on *P. lividus* embryos, 1:1000, 1:10,000 and 1:100,000, in volume. Higher concentrations (1:5, 1:10, 1:100) produced immediate block of embryo development at the first division and they were not considered in further analyses. The test was prepared starting from two mature females and one male of *P. lividus* collected in the Bay of Naples (Italy). Sea urchins were injected with 1 ml of 0.5 M KCl into the coelom through the soft derma around the mouthparts, to stimulate the contraction of gonads. They were vigorously shaken and females were placed with mouths up, over a 50 ml beaker, until the gametes were released into filtered (0.22 µm, Millipore) seawater, to facilitate the collection of eggs. Eggs were further rinsed three times with clean seawater to remove possible organic residuals (Chapman, 1995). Sperm were collected ‘dry’, using a Pasteur pipette and sucking over the surface of male gonopores, to avoid premature activation. The gametes obtained from each individual were conserved in plastic vessels until fertilization, when sub-samples of eggs were collected and added with a drop of sperm suspension. Egg activation was revealed by the elevation of the fertilization membrane within 40–80 s, appearing as a clear circle. Pools of embryos exhibiting percentages of fertilization lower than 95% were discarded. Pools exhibiting viable embryos were used for bioassays. To this end, groups of 500 embryos obtained from each replicate female were collected in duplicate and transferred into 5 ml multi-well dishes filled with appropriate dilutions of the cyanobacteria spent media, as specified above.

Four replicate tests were run at each concentration of the spent culture medium, plus four negative controls prepared using only seawater added with corresponding proportions of fresh *f/2* medium. The results were recorded at various time intervals and according to each concentration. In particular, multi-wells were inspected after 1 h under the inverted microscope to record the percentage of individuals showing normal cell division and, eventually, the presence of apoptosis hallmarks, such as blebbings. After 6 h and 24 h, 1 ml of egg suspension was fixed with the addition of a drop of 40% buffered formalin and replicates examined to record the percentage of individuals at the blastula, gastrula and prism stages, respectively. The percentage of individuals that were still at the first stages of divisions and those blocked or apoptotic was recorded as well. The remaining content of wells was fixed after 48 h and examined to record the percentage of normal plutei.

### Statistical analyses

Means and standard deviations of the readings obtained from various replicates, for each set of measurements, were organized in matrices. Raw data were analysed using factorial ANOVA that builds a linear model to include main-effects and interactions for categorical predictors. Multivariate (multiple continuous dependent variables) designs were analysed using Statistica version 10 (StatSoft Inc., Tulsa, OK, USA). The variance/covariance matrix of dependent variables was tested for normality and homogeneity of variances by the Kolmogorov–Smirnov and Levene's tests, respectively. Wilcoxon test was used to check the significance of differences between individual treatments. Other graphs and statistical analyses were computed using GraphPad Prism version 6.00 for Macintosh (GraphPad Software, La Jolla, CA, USA, www.graphpad.com).

### Ethical approval and consent to participate

All animal experiments were carried in accordance with the EU Directive 2010/63/EU and The Code of Ethics of the World Medical Association (Declaration of Helsinki) for animal experiments.
